# Recurrence and Outcomes of Lupus Nephritis After Renal Transplantation: Analysis of Nine Cases and Review of the Literature

**DOI:** 10.3390/jcm15051682

**Published:** 2026-02-24

**Authors:** Magdalena Morytko, Radosław Dziedzic, Weronika Pociej-Marciak, Mariusz Korkosz, Joanna Kosałka-Węgiel

**Affiliations:** 1 University Hospital in Kraków, Clinical Department of Rheumatology, Immunology and Internal Medicine, Jakubowskiego 2, 30-688 Kraków, Poland; m.morytko1@gmail.com (M.M.); radoslaw.dziedzic@doctoral.uj.edu.pl (R.D.); mariusz.korkosz@uj.edu.pl (M.K.); 2 Jagiellonian University Medical College, Doctoral School of Medical and Health Sciences, św. Łazarza 16, 31-530 Kraków, Poland; 3 Jagiellonian University Medical College, Division of Ophthalmology and Ocular Oncology, Department of Ophthalmology, Faculty of Medicine, Kopernika 38, 31-501 Kraków, Poland; weronika.pociej-marciak@uj.edu.pl; 4 University Hospital in Kraków, Department of Ophthalmology and Ocular Oncology, Kopernika 38, 31-501 Kraków, Poland; 5 Jagiellonian University Medical College, Department of Rheumatology and Immunology, Jakubowskiego 2, 30-688 Kraków, Poland

**Keywords:** end stage renal disease, kidney transplantation, lupus nephritis, prognosis, systemic lupus erythematosus

## Abstract

**Background and Objectives:** Recurrence of lupus nephritis (LN) after kidney transplantation is a major clinical concern in patients with systemic lupus erythematosus (SLE) who progress to end-stage renal disease (ESRD). Reported rates of post-transplant LN recurrence vary widely and are influenced by patient characteristics, immunosuppressive regimens, and indications for allograft biopsy. **Patients and Methods:** Medical records of adult LN patients treated at the University Hospital in Kraków, Poland, during the years 2012–2022 were retrospectively reviewed to identify individuals who progressed to ESRD and received a kidney transplant. Data collected included patient demographics as well as clinical, laboratory, transplant-related, and dialysis-related information. **Results:** Among 1039 patients with SLE, LN was diagnosed in 351 (33.8%), and 28 (8.0%) progressed to ESRD, of whom n = 9 (32.1%) underwent kidney transplantation. All patients received deceased-donor grafts, with a median time from ESRD to transplantation of 3 years (range 1–8) and a median post-transplant follow-up of 6 years (3–20). Maintenance immunosuppression consisted predominantly of glucocorticosteroids, mycophenolate mofetil, and tacrolimus in 77.8% of patients, with basiliximab induction was used in 2 of 2 patients with available data. Biopsy-proven LN recurrence occurred in 22.2% (2/9) of recipients. Graft loss was observed in 22.2% (2/9), while overall mortality reached 33.3% (3/9), including one peri-transplant death and one death due to infectious complications. Hematological manifestations were present in 100% of patients, hypercholesterolemia in 100%, and arterial hypertension in 88.9%, while anti-dsDNA antibodies were detected in 77.8%. LN relapse occurred despite standard immunosuppressive therapy and in the absence of consistent clinical or immunological predictors. **Conclusions:** LN recurrence occurred in 2 of 9 patients (22.2%). Patients with LN after kidney transplantation require careful long-term monitoring and individualized immunosuppressive management, considering baseline risk profile and relevant clinical with immunological factors.

## 1. Introduction

Systemic lupus erythematosus (SLE) is a chronic multisystem autoimmune disease characterized by a broad spectrum of clinical manifestations and variable organ involvement. Typical clinical features include inflammatory arthritis, cutaneous manifestations such as the malar (“butterfly”) rash, fatigue, photosensitivity, oral ulcers, and fever [1]. In addition to these common presentations, SLE may involve major organ systems, including the kidneys, cardiovascular system, lungs, and central nervous system, contributing substantially to disease-related morbidity and mortality [2]. Renal involvement is one of the most frequent and clinically significant manifestations of SLE and may constitute the initial presentation in approximately 7–31% of cases [3]. Over the course of the disease, lupus nephritis (LN) develops in an estimated 20% to 60% of patients [4]. Despite substantial advances in immunosuppressive therapy, the clinical course of LN remains highly heterogeneous. Data regarding the timing and risk of progression to end-stage renal disease (ESRD) are inconsistent, reflecting differences in study populations, treatment strategies, and duration of follow-up [5]. As stated in a meta-analysis by Tektonidou et al. [6] risks of ESRD in LN improved between the 1970s and the mid-1990s and then plateaued, with an increase in the late 2000s.

In fact, kidney transplantation is the treatment of choice for patients with LN who progress to ESRD, as it is associated with superior patient survival, improved quality of life, and lower complication rates compared with long-term dialysis [7,8]. Nevertheless, recurrence of LN in the renal allograft has long been recognized as a clinically relevant concern. Although recurrence disease is associated with an increased risk of allograft dysfunction, graft loss directly attributable to LN recurrence remains relatively uncommon [9]. Reported recurrence rates vary widely (2.5–54%), reflecting heterogeneity in patient populations, biopsy indications, immunosuppressive regimens, and histopathological assessment methods [10]. When indication biopsies are evaluated using light microscopy in combination with immunofluorescence and electron microscopy, recurrence rates of approximately 18–30% have been reported [11,12]. Even higher recurrence rates, ranging from 43% to 54%, have been observed in studies employing protocol biopsy strategies with similarly comprehensive histological evaluation, likely reflecting the detection of subclinical disease [13,14]. In contrast, other large cohort studies using different immunosuppressive protocols and biopsy practices have reported substantially lower recurrence rates, typically ranging from 0% to 11.3% [9,10].

Early studies assessing LN recurrence after kidney transplantation predominantly reflected immunosuppressive regimens based on cyclosporine and azathioprine, which were standard therapies at the time [7]. Over subsequent decades, post-transplant immunosuppressive strategies have evolved substantially in response to improvements in graft survival and reductions in rejection rates [7]. Tacrolimus progressively replaced cyclosporine as the primary calcineurin inhibitor beginning in the late 1990s and became the dominant agent in clinical practice by the late 2000s [15,16]. In parallel, mycophenolate mofetil was increasingly adopted from the mid-1990s onward, largely supplanting azathioprine as the preferred antimetabolite for maintenance immunosuppression [7,16]. Despite these major therapeutic advances, the impact of contemporary immunosuppressive regimens, particularly combinations of tacrolimus and mycophenolate mofetil, on the risk of LN recurrence in the renal allograft remains incompletely understood [14]. Therefore, we aimed to evaluate clinical outcomes in a Polish cohort of patients with LN who underwent kidney transplantation in the contemporary immunosuppressive era dominated by tacrolimus and mycophenolate mofetil. Both tacrolimus and mycophenolate mofetil are currently recommended as first-line agents for the treatment of LN itself, further underscoring the clinical relevance of evaluating their impact on post-transplant outcomes [14,17]. Specifically, we sought to assess patient and graft survival, determine the incidence of LN recurrence, and quantify the risk of acute and chronic allograft rejection. Given that most available data on post-transplant outcomes in LN originate from North American and Asian cohorts, this study also aims to address the relative paucity of evidence from Europe and to provide region-specific insights into transplant outcomes in this population.

## 2. Patients and Methods

### 2.1. Patient Population

A retrospective analysis was conducted using medical records of patients with SLE who received treatment at the University Hospital in Kraków, Poland, between 2012 and 2022. Patients were initially identified using the ICD-10 code M32. Only individuals who fulfilled the 2019 European League Against Rheumatism (EULAR) and the American College of Rheumatology (ACR) classification criteria were included [18].

First, we identified all patients diagnosed with LN, and within this cohort we screened for individuals who progressed to ESRD requiring dialysis. Progression from LN to ESRD was defined as the initiation of chronic renal replacement therapy (hemodialysis or peritoneal dialysis) or preemptive listing for kidney transplantation due to irreversible loss of kidney function. The diagnosis of LN was established through renal biopsy classified according to the ISN/RPS (International Society of Nephrology/Renal Pathology Society) criteria or, alternatively, based on clinical evidence of renal involvement, such as proteinuria and active urinary sediment, during a lupus flare [19]. This subgroup with ESRD was subsequently followed to evaluate outcomes related to kidney transplantation. For all included patients, we collected detailed demographic data, including sex and age, as well as comprehensive clinical and laboratory information. These encompassed the onset of SLE, the onset of LN, overall disease duration, the interval between SLE/LN diagnosis and the development of ESRD, coexisting comorbidities, history of miscarriages in women, therapeutic strategies used throughout the disease course, as well as, when applicable, the cause of death and the age at which death occurred.

The clinical features evaluated in this study comprised cutaneous manifestations, arthritis, serositis, hematologic disorders, organ-specific involvement of the kidneys, liver, nervous system, and respiratory system, as well as Raynaud’s phenomenon and lymphadenopathy. Detailed descriptions of the clinical variables and assessment methods have been presented in our previous publication [5]. Additionally, we collected data regarding post-transplant disease activity, including SLE exacerbations—with particular emphasis on renal flares—immunosuppressive regimens, graft function over time, and transplant-related complications such as acute rejection, infections, or graft loss.

Post-transplant recurrence of LN was defined as biopsy-proven LN in the kidney allograft. Kidney biopsies were performed exclusively for clinical indications (for-cause biopsies), such as unexplained deterioration of graft function, new-onset or worsening proteinuria, or active urinary sediment. Histopathological evaluation was used to confirm LN recurrence and to differentiate it from other causes of graft dysfunction. Competing etiologies, including acute rejection, calcineurin inhibitor toxicity, and infection, were systematically excluded based on histological findings, clinical assessment, laboratory data, drug trough levels, and microbiological testing, as appropriate. Protocol (surveillance) biopsies were not performed in asymptomatic patients.

Categorical variables were expressed as counts and percentages. Continuous variables were presented as medians with Q1–Q3 ranges.

The study received approval from the Bioethics Committee of the Jagiellonian University Medical College (approval no. 118.6120.21.2023; 15 June 2023) and was conducted in accordance with the principles of the Declaration of Helsinki.

### 2.2. Laboratory Testing

Routine laboratory methods were applied to assess complete blood counts, lipid panels, serum creatinine, and estimated glomerular filtration rate (eGFR), which was calculated using the MDRD formula. Urinary evaluation included measurement of 24 h proteinuria and microscopic analysis of urinary sediment. Antinuclear antibodies (ANA) were detected by indirect immunofluorescence using HEp-2 cells, while specific autoantibodies were identified by ELISA or line blot assays. Anti-double-stranded DNA antibodies were measured using the *Crithidia luciliae* immunofluorescence assay, and complement levels were determined by nephelometry. Assessment of hypercoagulability included lupus anticoagulant testing and quantification of anti-cardiolipin and anti–β2-glycoprotein I antibodies (IgG and IgM).

## 3. Results

### 3.1. General Characteristics of the Analyzed Cohort of Lupus Nephritis Patients After Renal Transplantation

LN was confirmed in 351 of 1039 patients with SLE (33.78%). ESRD developed in 28 of 351 LN patients (7.98%). Kidney transplantation was performed in only 9 of these 28 patients (32.14%), including 6 Caucasian women and 3 Caucasian men. LN was diagnosed concurrently with SLE onset in seven cases, one case was classified as early LN (diagnosed one year after SLE onset), and in one case the timing of LN diagnosis was unknown. The median age at SLE onset was 25 years (15–41.5 years), and the median age at LN diagnosis was 26.5 years (16.5–41.5 years). Regarding LN flares, six patients experienced only one flare, one patient had two flares (Patient No. 5), one patient had three flares (Patient No. 9), and in one case the number of flares was unknown (Patient No. 1). Kidney biopsy was performed in only four cases: class IV was the most frequent finding (two cases, Patients No. 6 and No. 9), while classes V and VI were each observed in one case (Patients No. 5 and No. 8, respectively). There was no family history of SLE; however, in two cases a positive family history of rheumatoid arthritis was reported (mother of Patient No. 4 and great-aunt of Patient No. 9). Detailed patient characteristics are presented in Table 1.

### 3.2. Clinical Manifestations

The most prevalent clinical manifestations except for kidney involvement, were hematological abnormalities (n = 9, 100%), and constitutional symptoms (n = 6, 66.67%). A detailed analysis revealed that certain symptoms were present in all SLE patients diagnosed with ESRD after kidney transplantation, specifically lymphopenia and anemia. Additionally, most patients, 5 of 9 (55.56%), exhibited arthralgia, leukocytopenia, and thrombocytopenia. Lupus malar rash, arthritis, and pleural effusion were observed in 4 out of 9 patients (44.44%). In 3 out of 9 patients (33.33%), fatigue/weakness and weight loss were observed. In 2 out of 9 cases (22.22%) were observed myalgias, pericardial effusion and haemolytic anaemia. Only single case (11.11%) presented with fever, discoid rash, alopecia, oral and/or nasal ulcers, pericarditis, and Raynaud’s phenomenon. None of the patient presented with lymphadenopathy, urticaria, photosensitivity, neurological symptoms, lung involvement and lupoid hepatitis. Detailed distributions of systemic symptoms in the ESRD-SLE study group following kidney transplantation are presented in Table 2.

### 3.3. Comorbidities

Hypercholesterolemia was the most common comorbidity and was confirmed in all patients (n = 9, 100%). Arterial hypertension was diagnosed in all but one patient (n = 8, 88.89%), except for Patient No. 3. Diabetes mellitus and atrial fibrillation were each observed in one patient (Patient No. 7). None of the patients had hypothyroidism, hyperthyroidism, peripheral artery disease, malignancy, or a history of myocardial infarction.

### 3.4. Autoantibody Profiles

Antinuclear antibodies (ANA) were detected in all patients, with titers ranging from 1:640 to 1:20,480. Among SLE patients after kidney transplantation, the most frequently detected antibodies were anti–double-stranded DNA (anti-dsDNA), present in 7 of 9 patients (77.78%), except for Patients No. 5 and No. 7. Anti-SSA antibodies were detected in three patients (Patients No. 3, No. 4, and No. 5). In addition, Patient No. 1 tested positive for anti-histone and anti-nucleosome antibodies, Patient No. 3 for anti-SSB antibodies, and Patient No. 8 for anti-RNP antibodies. All patients were negative for anti-Sm antibodies and antineutrophil cytoplasmic antibodies (ANCA).

Only one patient (Patient No. 5) tested positive for antiphospholipid antibodies (aPLA), limited to lupus anticoagulant. This patient fulfilled the 2023 EULAR/ACR classification criteria for antiphospholipid syndrome based on the presence of lupus anticoagulant and a history of deep venous thrombosis and pulmonary embolism.

### 3.5. Immunosuppressive Treatment

Glucocorticosteroids were administered in all nine patients (100%). Mycophenolate mofetil was used in all patients except one (Patient No. 2). Azathioprine was administered in seven patients, except for Patients No. 7 and 9. Cyclophosphamide was used in five patients (Patients No. 1, No. 4, No. 5, No. 8, and No. 9). Cyclosporine A was administered in three patients (Patients No. 1, 5, and 7), and plasmapheresis was performed in three patients (Patients No. 1, No. 4, and No. 6). Chloroquine or hydroxychloroquine was used in three patients only (Patients No. 1, No. 4, and No. 9). No other immunosuppressive therapies, including methotrexate, sulfasalazine, belimumab, anifrolumab, or rituximab, were administered in the analyzed cohort.

### 3.6. Characteristics of Kidney Recipients with Lupus Nephritis After Kidney Transplantation

**Figure 1:**
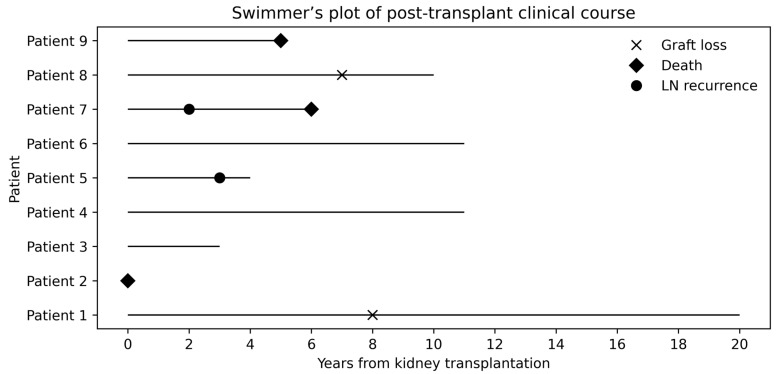
Swimmer’s plot of individual post-transplant clinical courses in analyzed lupus patients. Swimmer’s plot illustrating each lupus patient’s clinical journey from kidney transplantation to lupus nephritis recurrence, graft loss, or death. Each horizontal bar represents the duration of follow-up from transplantation. Symbols indicate the timing of lupus nephritis recurrence (circle), graft loss (cross), and death (diamond). Abbreviations: LN—lupus nephritis.

The median time from ESRD to kidney transplantation was 3 years (1.5–5.5 years), and all patients received kidneys from deceased donors. The median duration of post-transplant follow-up was 6 years (3.5–11 years). Patient No. 2 died in the peri-transplant period and was therefore excluded from post-transplant follow-up analyses. Basiliximab was administered as induction therapy in patients No. 3 and No. 8; data regarding induction therapy in the remaining patients were unavailable. All remaining patients, except Patient No. 2 and No. 7, received systemic glucocorticoids, mycophenolate mofetil, and tacrolimus as maintenance immunosuppressive therapy following kidney transplantation. Patient No. 7 was treated with systemic glucocorticoids, mycophenolate mofetil, and cyclosporine A. Recurrence of LN was confirmed by kidney biopsy in two patients (Patient No. 5 and No. 7; 22.22%). Graft loss occurred in two cases (Patient No. 1 and No. 8). In Patient No. 1, graft failure resulted from chronic antibody-mediated rejection progressing to end-stage graft dysfunction, whereas the etiology in Patient No. 8 remained undetermined. However, Patient No. 1 subsequently underwent a second kidney transplantation, and during the subsequent 6-year follow-up there was no recurrence of LN and no further graft loss. Overall, three patients (33.33%) died during the study period: Patient No. 2 in the peri-transplant period, Patient No. 7 due to infectious complications (pneumonia) complicated by multiorgan failure, and Patient No. 9 from an unknown cause. Individual post-transplant clinical courses of patients with lupus nephritis are summarized in Figure 1, which presents a swimmer’s plot illustrating the timing of lupus nephritis recurrence, graft loss, and death in relation to kidney transplantation. Detailed post-transplant clinical characteristics, including donor type, immunosuppressive therapy, and follow-up duration, are provided in Table 3.

## 4. Discussion

The study provides insights into the characteristics of a single-center cohort of patients with LN who underwent kidney transplantation with post-transplant outcomes. To our knowledge, this study represents one of the most extensive analyses of Polish patients with SLE, addressing diverse disease characteristics in the context of kidney transplantation.

Reported estimates suggest that the five-year probability of progression to ESRD among patients with LN ranges widely [20]. In contrast, only 8% of LN patients in our cohort progressed to ESRD. This discrepancy may reflect differences in cohort characteristics, duration of follow-up, or the presence of incomplete or missing data [21,22]. Therefore, the results should be approached with caution in their interpretation. In our study, only 9 patients with LN ultimately underwent kidney transplantation, representing 32.14% of LN patients with ESRD based on our entire LN cohort. This proportion is substantially lower than that reported in large LN-ESRD cohorts, in which nearly three-quarters of patients receive a kidney transplant [23]. This discrepancy may be related to differences between countries or to a relatively short duration of follow-up after the onset of ESRD in the non-transplanted group.

Demographic analysis revealed that the cohort comprised six women and three men; however, it is well established that SLE predominantly affects women, who represent approximately 90% of all cases [2]. Therefore, the higher observed frequency of kidney transplantation in women may be partly relative, reflecting the underlying sex distribution of SLE rather than a truly increased female-specific risk. Consistent with our findings, Brilland et al. [23] reported that the majority of kidney transplantation procedures were performed in women. Next, the median age at LN onset in the cohort that underwent kidney transplantation during follow-up was 26.5 years. Moreover, this finding is consistent with studies comparing pediatric and adult SLE populations, in which ESRD is diagnosed nearly three times more frequently in the pediatric group [24].

It is well established that kidney transplantation represents a viable therapeutic option for patients with LN-associated ESRD [25,26]. Nevertheless, patients with LN who undergo kidney transplantation remain susceptible to multiple post-transplant complications, including graft rejection, infections, and disease relapse. In our cohort, biopsy-proven recurrence of LN was identified in 2 out of 9 patients (22.22%), a prevalence substantially higher than the 2.44% reported by Contreras et al. [9] in large registry-based data. Consistent with previous studies, female sex, younger age (<33 years), and black race have been identified as independent risk factors for recurrence LN [9]. Notably, neither of the two patients with recurrence LN in our cohort exhibited these established risk factors, except for age at recurrence in patient 7 (which is unknown). Previous studies have demonstrated that post-transplant LN recurrence may also occur in patients without classical high-risk characteristics, underscoring the need for vigilant post-transplant monitoring in all patients with SLE, regardless of baseline risk profile. According to emerging reports, subclinical, biopsy-detected recurrence of LN is more common than previously recognized is as high as 43–54% [27,28]. The silent recurrence is often associated with increased proteinuria, positive lupus anticoagulant status, and the use of living-donor grafts [27]. In our cohort, kidney biopsies were performed only in patients 5 and 7 who developed clinical signs of renal involvement after kidney transplantation, as routine surveillance biopsies were not conducted in asymptomatic patients. According to published evidence, the decision to perform post-transplant kidney biopsy should be individualized, based on the patient’s clinical presentation and risk profile.

Given the limited number of patients with recurrent LN following kidney transplantation in our cohort, we did not propose a formal post-transplant follow-up or management algorithm based solely on our data. However, available evidence supports vigilant monitoring of all kidney transplant recipients with SLE, including regular clinical and laboratory assessment of renal function, proteinuria, and serological markers, with timely for-cause biopsy in the context of graft dysfunction. Subclinical, biopsy-proven recurrence of LN has been reported in more than half of SLE transplant recipients undergoing surveillance biopsies, emphasizing the need for individualized surveillance strategies beyond routine clinical follow-up [27,29].

The most frequently observed clinical manifestations in our cohort, apart from kidney involvement, were hematological abnormalities, which were present in all patients, and general (constitutional) symptoms, diagnosed in six cases (66.67%). Similarly to our findings, the majority of studies report anemia, leucopenia, and thrombocytopenia in at least 36.4% of patients experiencing SLE relapse following kidney transplantation [29,30]. Given that hematological abnormalities are common in both SLE and ESRD, their etiology in post-transplant patients is likely complex and multifactorial, including autoimmune cytopenias, ESRD-related factors, and drug-induced effects [31,32]. Mild extrarenal manifestations, such as malar rash, arthralgia, and myalgia, were also frequently observed in our cohort, with prevalence of 44.44%, 55.56%, and 22.22%, respectively. In fact, kidney disease can manifest with ocular symptoms, so comprehensive patient assessment is essential [33]. Our findings are in line with those reported by Moroni et al. [34], who observed constitutional, mucocutaneous, and joint manifestations in 20% of patients, with effective symptom control achieved through corticosteroid therapy.

Pathogenic autoantibody production is a hallmark of SLE and plays a key role in the development of LN [35]. Anti-dsDNA antibodies are strongly associated with renal involvement in SLE, consistent with our previous observations demonstrating significantly higher anti-dsDNA levels in the LN cohort compared with non-LN SLE patients [36]. Furthermore, in our single-center retrospective study comparing early- and delayed-onset LN, delayed-onset LN was characterized by a higher prevalence of anti-dsDNA antibodies [5]. Current evidence suggests that markedly elevated anti-dsDNA titers, both before and after kidney transplantation, are associated with an increased risk of disease flares [29]. In the present cohort, 77.78% of patients tested positive for anti-dsDNA antibodies. Notably, patients 5 and 7, who experienced LN recurrence, had negative anti-dsDNA profiles, suggesting that disease recurrence may occur independently of classical serological markers and may reflect heterogenous post-transplant pathogenic mechanisms. Previous studies support the diagnostic and prognostic relevance of anti-dsDNA antibodies, while acknowledging their limitations in predicting post-transplant disease activity. Anti-Ro (SSA) antibodies are frequently detected in several autoimmune diseases, including Sjögren syndrome, SLE, systemic sclerosis, and myositis [37]. In SLE, their prevalence is estimated to range from approximately 40% to 90% [38]. Although associations between anti-SSA antibodies and cutaneous manifestations, sicca symptoms, and hematological abnormalities in SLE are well established, evidence regarding their pathogenic role in LN remains inconsistent [38,39]. In our cohort, anti-SSA antibodies were detected in 33.33% of patients, which falls within the wide range of prevalence reported in LN [36,40]. All patients in our cohort tested negative for anti-Sm antibodies, in contrast to reports demonstrating higher anti-Sm prevalence in LN [41]. This discrepancy may be partially explained by racial differences, as anti-Sm antibodies are more commonly observed in African American populations [42]. ANCA are present in a subset of patients with SLE, with a reported prevalence of 16.4% in large European cohorts [43]. Emerging evidence suggests an association between ANCA positivity at the time of transplantation and chronic graft dysfunction, as demonstrated in pediatric patients with childhood-onset ANCA-associated vasculitis [44]. However, data on the correlation between ANCA status and graft outcomes in patients with LN following kidney transplantation remain limited, and ANCA positivity has not yet been established as a prognostic factor in this population. In our cohort, no patients tested positive for ANCA, limiting further evaluation of its clinical significance in this population. Emerging evidence suggests that ANCA status may be of potential clinical relevance; however, its prognostic value in LN transplant recipients has not yet been established.

Several studies have emphasized the importance of transplant timing in patients with LN, as prolonged exposure to pre-transplant dialysis may increase the risk of graft failure [45,46]. In our cohort, the median time from ESRD to kidney transplantation was 3 years (1.5–5.5 years), which aligns with observations from previous studies [29,47,48]. Furthermore, preemptive transplantation has been associated with superior patient and graft survival and should be considered whenever feasible [29,49]. However, none of the patients in our cohort underwent preemptive transplantation, likely reflecting challenges related to timely referral and donor availability. Collectively, these findings underscore the importance of optimizing transplant timing to improve overall patient outcomes and highlight the need for further investigation to identify the most effective therapeutic strategies.

Post-transplant immunosuppressive therapy in patients with LN generally follows standard protocols based on a calcineurin inhibitor, most commonly tacrolimus, in combination with mycophenolate mofetil and glucocorticoids [50,51]. In fact, recent guidelines (KDIGO, ACR, EULAR) no longer require complete serologic remission before proceeding with kidney transplantation in patients with LN [14,52,53]. This shift in clinical practice further underscores the relevance of contemporary cohorts such as ours for informing real-world post-transplant outcomes. The preferential use of mycophenolate mofetil is supported by its proven efficacy in both induction and maintenance treatment of severe LN, as well as its established role in preventing allograft rejection following kidney transplantation [54]. Accordingly, mycophenolate mofetil was used in all patients in our cohort except one, who died in the peri-transplant period before initiation of maintenance immunosuppression. Cyclophosphamide, despite its less favorable side-effect profile, remains a standard induction therapy for severe LN and was administered to five patients in our cohort. This therapy is preferentially used in patients with high disease activity or progressive renal involvement and has been associated with high remission rates. Glucocorticoids remain a cornerstone of both LN treatment and post-transplant immunosuppression and were administered to all patients in our cohort in accordance with standard transplant protocols [55,56]. Two patients (patient No 3 and No 8) received additional basiliximab induction therapy alongside standard immunosuppression, reflecting that its use is determined by individual immunological risk assessment. However, evidence regarding the benefit of basiliximab induction therapy is inconsistent, with several studies reporting no significant advantage in terms of acute rejection or graft loss compared with no induction in patients with low immunological risk [57,58].

It remains unclear whether the risks of graft failure and patient mortality differ between LN recipients and non-LN cohorts. Data from the United States Renal Data System (USRDS) and several single-center studies have reported comparable graft and patient survival outcomes between the two groups. However, a recent systematic review suggests that LN may be associated with an increased risk of graft failure and mortality [34,59,60]. During the follow-up period of our study, 3 out of 9 patients (33.33%) died: one in the peri-transplant period, one due to infectious complications, and one from an unknown cause. Although the small cohort size limits the generalizability of these findings, mortality rates in our cohort are somewhat higher than those reported in larger studies. Nevertheless, emerging evidence indicates that kidney transplantation in patients with LN is generally associated with favorable long-term outcomes, with reported 5-year survival rates exceeding 80–90% in most studies [61,62,63]. According to previous cohort studies and systematic reviews, infectious complications and cardiovascular events remain the most frequent causes of death among LN patients following kidney transplantation [64,65]. In a cohort of 99 LN transplant recipients, infections and cardiovascular disease accounted for 30.8% and 38.5% of deaths with a functioning graft, respectively, which is consistent with findings from other reports [66,67]. Taken together, the causes of mortality in patients with LN are comparable to those observed in patients with ESRD due to other etiologies, apart from a higher infection-related mortality in the LN cohort [65,68].

Importantly, all patients in our cohort received deceased-donor kidney transplants, likely reflecting both systemic and sociocultural factors in Poland [69]. The national transplant system, based on presumed consent for organ donation, together with limited public awareness of living donation, restrictive legal regulations, and concerns regarding donor safety, facilitates deceased-donor transplantation [70]. Consequently, living-donor transplants account for only about 2–3% of all kidney transplantation procedures performed annually in Poland [70]. In contrast, reports from international LN cohorts indicate substantially higher rates of living-donor kidney transplantation, ranging from 27% to 66% [47,71]. This reliance on deceased-donor grafts in our cohort may have implications for post-transplant outcomes and should be considered when interpreting the results, given the generally less favorable graft and patient survival associated with deceased-donor transplantation [56].

Several limitations of this study should be acknowledged. First, the retrospective study design carries an inherent risk of bias related to data acquisition and patient selection. In addition, the number of patients with LN following kidney transplantation was relatively small; however, given the rarity of this complication, the findings remain clinically meaningful. Another important limitation is the small number of patients with recurrent LN after kidney transplantation, which precluded the development of an evidence-based follow-up or management algorithm derived from our cohort. As this was a single-center study, the applicability of the results to broader and more heterogeneous populations may be limited. Importantly, the observed mortality rate among patients with recurrence LN should be interpreted with caution, as it may appear disproportionately high due to the very small sample size, which precludes robust statistical analysis. Furthermore, due to the retrospective design and the scope of data collection, overall mortality data for all kidney transplant recipients at our center were not available and therefore could not be analyzed or used for contextual comparison. Another limitation of this study is the absence of patient-reported outcomes, including quality-of-life measures, which would have offered a more comprehensive assessment of patient well-being and the overall impact of both the disease and its treatment. In addition, some of the observed associations may be coincidental and should not be interpreted as causal relationships.

## 5. Conclusions

This study provides valuable insights into the recurrence and outcomes of LN after renal transplantation based on a single-center cohort. Taken together, careful risk stratification, vigilant post-transplant monitoring, and individualized immunosuppressive management remain essential for improving long-term outcomes. Although established risk factors for LN recurrence should be considered during transplant evaluation and follow-up, previous studies indicate that LN recurrence may occur independently of currently recognized clinical and immunological predictors. Notably, the rate of disease recurrence in our cohort was substantial, underscoring the need for ongoing vigilance regardless of baseline risk profile. Importantly, all recipients received deceased-donor grafts, which may have influenced post-transplant outcomes and should be considered when interpreting these findings. Prospective, multicenter studies are warranted to better define predictors of LN recurrence and to optimize post-transplant management strategies in this population.

## Figures and Tables

**Table 1:** Demographic, clinical, and histopathological characteristics of lupus nephritis patients after renal transplantation.

	No. 1	No. 2	No. 3	No. 4	No. 5	No. 6	No. 7	No. 8	No. 9
Sex (female/male)	F	F	F	F	M	F	M	M	F
Ethnicity	C	C	C	C	C	C	C	C	C
Age at the SLE onset, years	16	13	39	25	17	27	54	44	14
Age at LN diagnosis, years	16	NA	39	26	17	27	54	44	14
Age at last visit, years	36	35	47	35	32	38	64	57	29
Number of LN flares	U	1	1	1	2	1	1	1	3
Histological class	U	U	U	U	V	IV	U	VI	IV
Positive family history	N	N	N	Y	N	N	N	N	Y

Abbreviations: C—Caucasian; F—female; LN—lupus nephritis; M—male; N—no; NA—not available; SLE—systemic lupus erythematosus; U—unknown; Y—yes.

**Table 2:** Systemic involvement in patients with systemic lupus erythematosus after kidney transplantation.

Clinical Manifestations	LN Patients After Kidney Transplantation n = 9
Constitutional symptoms, n (%)	6 (66.67%)
Fever, n (%)	1 (11.11%)
Fatigue/weakness, n (%)	3 (33.33%)
Myalgias, n (%)	2 (22.22%)
Weight loss, n (%)	3 (33.33%)
Lymphadenopathy, n (%)	0 (0%)
Mucocutaneous manifestations, n (%)	5 (55.56%)
Lupus malar rash, n (%)	4 (44.44%)
Discoid rash, n (%)	1 (11.11%)
Urticaria, n (%)	0 (0%)
Alopecia, n (%)	1 (11.11%)
Oral and/or nasal ulcers, n (%)	1 (11.11%)
Photosensitivity, n (%)	0 (0%)
Joint manifestations, n (%)	5 (55.56%)
Arthritis, n (%)	4 (44.44%)
Arthralgia, n (%)	5 (55.56%)
Serositis, n (%)	5 (55.56%)
Pleural effusion, n (%)	4 (44.44%)
Pericardial effusion, n (%)	2 (22.22%)
Pericarditis, n (%)	1 (11.11%)
Hematological manifestations, n (%)	9 (100.0%)
Leucopenia ^#^, n (%)	5 (55.56%)
Lymphopenia ^$^, n (%)	9 (100.0%)
Anemia ^&^, n (%)	9 (100.0%)
Hemolytic anemia *, n (%)	2 (22.22%)
Thrombocytopenia ^@^, n (%)	5 (55.56%)
Kidney involvement, n (%)	9 (100%)
Neurological signs, n (%)	0 (0%)
Central nervous system involvement, n (%)	0 (0%)
Peripheral nervous system involvement, n (%)	0 (0%)
Raynaud’s phenomenon, n (%)	1 (11.11%)
Lung involvement, n (%)	0 (0%)
Interstitial lung disease, n (%)	0 (0%)
Diffuse alveolar haemorrhage, n (%)	0 (0%)
Lupoid hepatitis, n (%)	0 (0%)

Abbreviations: ^&^: hemoglobin ≤12 g/dL in women or ≤13.5 g/dL in men, or diagnosis based on medical history; ^#^: leukocyte count <4000/µL or diagnosis based on medical history; ^$^: lymphocyte count <1500/µL or diagnosis based on medical history; *: anemia with a positive direct Coombs test, decreased haptoglobin levels, or diagnosis based on medical history; ^@^: platelet count <100,000/µL or diagnosis based on medical history; LN: lupus nephritis; n: number.

**Table 3:** Post-transplant characteristics of kidney recipients with lupus nephritis.

	No. 1	No. 2	No. 3	No. 4	No. 5	No. 6	No. 7	No. 8	No. 9
Donor type	D	D	D	D	D	D	D	D	D
Time from ESRD to kidney transplantation, years	6	3	1	1	2	5	4	8	2
Maintenance immunosuppression	GC + MMF + TAC	NA	GC + MMF + TAC	GC + MMF + TAC	GC + MMF + TAC	GC + MMF + TAC	GC + MMF + CsA	GC + MMF + TAC	GC + MMF + TAC
Induction therapy	NA	NA	Basiliximab	NA	NA	NA	NA	Basiliximab	NA
LN recurrence (biopsy-proven)	N	NA	N	N	Y	N	Y	N	N
Graft loss	Y	NA	N	N	N	N	N	Y	N
Death	N	Y	N	N	N	N	Y	N	Y
Follow-up since kidney transplantation, years	20	0	3	11	4	11	6	10	5

Abbreviations: CsA—cyclosporine A; D—deceased; ESRD—end-stage renal disease; GC—glucocorticoids; LN—lupus nephritis; MMF—mycophenolate mofetil; NA—not available; TAC—tacrolimus.

## Data Availability

The data presented in this study are available on reasonable request from the corresponding author.
